# Bidirectional Associations Between Physical Activity, Sedentary Behavior, and Daily Symptoms in Patients With Chronic Obstructive Pulmonary Disease: Longitudinal Observational Study

**DOI:** 10.2196/65653

**Published:** 2025-10-22

**Authors:** Banchia Palmen, Zjala Ebadi, Maarten van Herck, Yvonne M J Goërtz, Qichen Deng, Melissa S Y Thong, Chris Burtin, Jeannette B Peters, Roy T M Sprooten, Erik W M A Bischoff, Emiel F M Wouters, Mirjam A G Sprangers, Jan H Vercoulen, Sarah Houben-Wilke, Anouk W Vaes, Daisy J A Janssen, Martijn A Spruit

**Affiliations:** 1 Institute of Nutrition and Translational Research in Metabolism, Care and Public Health Research Institute, Faculty of Health, Medicine and Life Sciences, Maastricht University, Maastricht, The Netherlands; 2 Department of Research and Development, Ciro Centre of Expertise for Chronic Organ Failure, Hornerheide 1, Horn, 6085 NM, The Netherlands, 31 0475587600; 3 Department of Medical Psychology, Radboud University Nijmegen Medical Centre, Nijmegen, The Netherlands; 4 Department of Pulmonary Diseases, Radboud University Medical Centre, Nijmegen, The Netherlands; 5 Living Lab in Ageing and Long-Term Care, Maastricht University, Maastricht, The Netherlands; 6 Department of Health Services Research, Care and Public Health Research Institute, Faculty of Health, Medicine and Life Sciences, Maastricht University, Maastricht, The Netherlands; 7 Amsterdam UMC location University of Amsterdam, Medical Psychology, Amsterdam, The Netherlands; 8 Unit of Cancer Survivorship, Division of Clinical Epidemiology and Aging Research, German Cancer Research Center (DKFZ), Heidelberg, Germany; 9 REVAL - Rehabilitation Research Center, BIOMED - Biomedical Research Institute, Faculty of Rehabilitation Sciences, Hasselt University, Diepenbeek, Belgium; 10 Department of Respiratory Medicine, Maastricht University Medical Center, Maastricht, The Netherlands; 11 Department of General Practice, Erasmus MC, Rotterdam, The Netherlands; 12 Department of Health Services Research and Department of Family Medicine, Care and Public Health Research Institute, Faculty of Health, Medicine and Life Sciences, Maastricht University, Maastricht, The Netherlands; 13 Department of Expertise and Treatment, Proteion, Horn, The Netherlands; 14 Department of Respiratory Medicine, Institute of Nutrition and Translational Research in Metabolism, Faculty of Health, Medicine and Life Sciences, Maastricht University, Maastricht, The Netherlands

**Keywords:** COPD, chronic obstructive pulmonary disease, ecological momentary assessment, accelerometry, physical activity, sedentary behavior, symptom assessment

## Abstract

**Background:**

Questionnaire-based symptom assessment may introduce recall bias and lacks bidirectional exploration. This is particularly relevant, given the unclear direction of the associations between physical activity (PA), sedentary time (ST), and symptoms in patients with chronic obstructive pulmonary disease (COPD). Understanding these associations could inform symptom management strategies and improve patient quality of life.

**Objective:**

This study aimed to investigate the direction of the association between PA, ST, and symptoms in patients with COPD using accelerometry and ecological momentary assessment (EMA).

**Methods:**

A subsample from the FAntasTIGUE study answered 8 randomly timed EMA questionnaires daily for 5 days. Ten symptoms were rated on a 7-point Likert scale: “I feel relaxed, short of breath, energetic, cheerful, insecure, irritated, satisfied, anxious, tired, and mentally fit.” Concurrently, step count and ST were measured using the ActiGraph GT9X Link placed on the right hip. Step count and ST 15 and 30 minutes pre- and post-EMA were used in multilevel models, controlled for pre-EMA steps and ST, and the previous EMA score. Significant confounders were used as covariates, and patient ID was used as random intercept.

**Results:**

Thirty-four patients (19/34, 56% men, mean age 66, SD 7 years; forced expiratory volume in 1 second 52±20% predicted; 1035 EMA responses) were included. Feeling more relaxed was associated with a higher step count 15 minutes post-EMA (β=5.1; 95% CI 0.9 to 10.1; *P*=.046). Conversely, higher step count 15 and 30 minutes pre-EMA was associated with feeling less relaxed (β=−5.2×10^−4^; 95% CI −9.7×10^−4^ to −7.0×10^−5^; *P*=.02; and β=−3.2×10^−4^; 95% CI −5.6×10^−4^ to −7.9×10^−5^; *P*=.009), more short of breath (β=8.5×10^−4^; 95% CI 4.7×10^−4^ to 1.2×10^−3^; *P*<.001; and β=4.6×10^−4^; 95% CI 2.6×10^−4^ to 6.6×10^−4^; *P*<.001), and tired (β=5.1×10^−4^; 95% CI 7.2×10^−5^ to 9.4×10^−4^; *P*=.02; and β=2.9×10^−4^; 95% CI 5.3×10^−5^ to 5.2×10^−4^; *P*=.02). Higher ST 15 and 30 minutes pre-EMA was associated with feeling more anxious (β=1.7×10^−4^; 95% CI 1.7×10^−5^ to 3.2×10^−4^; *P*=.03; and β=8.5×10^−5^; 95% CI 2.5×10^−6^ to 1.7×10^−4^; *P*=.04).

**Conclusions:**

A bidirectional association of feeling relaxed with PA was found in patients with COPD. Higher step count was related to feeling more short of breath and tired, whereas higher ST was associated with heightened anxiety.

## Introduction

Chronic obstructive pulmonary disease (COPD) is a heterogeneous chronic lung condition characterized by shortness of breath, cough, and/or sputum production. This is, at least partly, due to abnormalities of the airways or alveoli that cause persistent airflow obstruction [1]. The heterogeneity of COPD manifests in variations in disease progression, symptom burden, and response to interventions, requiring personalized care [2]. In addition to respiratory symptoms, patients with COPD frequently experience physical symptoms such as fatigue, alongside psychological symptoms such as depressed mood and anxiety [3,4]. Moreover, they are physically less active and spend more time sitting and lying down compared to healthy controls, contributing to disease progression and poorer health outcomes [5-9].

Both the number of steps per day and sedentary time (ST) are linked to the transient exacerbation of symptoms in patients with COPD [7,10,11]. However, the direction of these associations is unclear [10-13]. For example, while physical activity (PA) may enhance feeling short of breath immediately, during, or after PA, it is also possible that worsening symptoms lead to a decline in PA and increased ST. This short-term bidirectional relationship suggests a potential vicious cycle of physical inactivity and symptom exacerbation. A deeper understanding of these associations is essential for developing targeted interventions aimed at interrupting this cycle and improving symptom management [7,8,14]. Increasing PA and reducing ST have been shown to improve symptoms and health-related quality of life in patients with COPD and may even improve survival [7,8,13,14].

Traditional symptom assessment methods typically involve clinical interviews and questionnaires asking about symptom burden 1 to 2 weeks retrospectively, which can be subject to recall bias [15,16]. This creates a challenge, considering that patients with COPD experience variability in PA, ST, and symptoms within the day and from day to day [17-19]. To address this limitation, ecological momentary assessment (EMA) emerges as a feasible method to assess symptom experience over time. EMA is defined as ‘repeated sampling of people’s current thoughts, emotions, behavior, physiological states, and context, in their natural environment, typically (but not necessarily) via electronic wearable devices*’* [15,16,20]. Compared to traditional assessment methods, EMA minimizes recall bias, and ecological validity is maximized because events are monitored as close in time to their occurrence as possible [20-24]. EMA has been validated across multiple study populations, but the use of EMA for capturing symptoms in patients with COPD is still limited, and further examination of its psychometric properties is needed [25-28]. It offers a less cumbersome and cost-effective alternative for assessing multiple symptoms, making it suitable for COPD research [21,22,29]. Combining information collected with EMA and from continuous PA tracking such as accelerometry may reveal the complex bidirectional relationship between PA, ST, and symptoms. The ActiGraph accelerometer (ActiGraph Corp, Pensacola, FL, USA) has been validated for measuring PA and ST in patients with COPD, demonstrating good reliability. Its use in COPD research has been supported because of its sensitivity in detecting variations in PA and ST, making it suitable for real-world monitoring of activity patterns. Limited research is available where the temporal associations between PA, ST, and symptoms in patients with COPD are explored using real-time assessment [29].

Therefore, the main objective of this study was to investigate the direction of the association between PA and ST measured with accelerometry and physical and psychological symptoms measured with EMA in patients with COPD. It is hypothesized that PA would be associated with subsequent increases in symptoms such as breathlessness and fatigue in the short term, while worsening symptoms would predict subsequent reductions in PA and increasing ST. In addition, we aimed to explore whether the strength of these associations differed across the 10 symptoms assessed in this study. By addressing these gaps in knowledge, this study contributes to a more nuanced understanding of how daily activity patterns and symptom fluctuations interact in COPD, with potential implications for personalized intervention strategies.

## Methods

### Design

These analyses were a part of a multicenter, longitudinal, observational study in patients with COPD investigating physical, systemic, psychological, and behavioral factors associated with precipitation and/or perpetuation of fatigue (FAntasTIGUE study) [30]. The prevalence of fatigue and its associated factors in patients with COPD participating in the FAntasTIGUE study has been published before [3,31].

### Ethical Considerations

The study was approved by the Medical Research Ethics Committee United, Nieuwegein, The Netherlands (R17.036/NL60484.100.17) and was registered in the Dutch Trial Register (NTR6933). Written informed consent was obtained from all study participants. Participation was entirely voluntary, and participants could withdraw at any time without consequence. Participants did not receive any financial compensation. Data were deidentified before analysis.

### Participants

Patients were recruited between 2018 and 2021 via the pulmonary consultation at the outpatient clinics of the Department of Respiratory Medicine in Maastricht and the Department of Pulmonary Diseases of the Radboud University Medical Center in Nijmegen and via general practitioners (Research Network Family Medicine Maastricht [32] and the Academic General Practitioner Network in the Nijmegen region). In addition, patients with COPD who attended a meeting for patients with chronic lung diseases (cfr. *longpunt*) in Maastricht or Nijmegen and patients recruited from primary and secondary care that previously participated in the Chance Study (NTR3416) and had indicated on the informed consent form that they agreed to be approached for follow-up research were invited to participate in the FAntasTIGUE study. Patients had to meet the following criteria for inclusion: (1) a diagnosis of COPD according to the Global Strategy for the Diagnosis, Management and Prevention of COPD (Global Initiative for Chronic Obstructive Lung Disease [GOLD], grade 1A–4D), (2) no exacerbation-related hospitalization less than 4 weeks preceding enrollment, (3) no use of oral corticosteroids and/or antibiotics less than 4 weeks preceding enrollment, and (4) provided written informed consent.

A subsample of the first 60 patients who were interested in participating in the EMA study had to meet these extra inclusion criteria: (1) access to the internet at home (Wi-Fi) and (2) able to operate an iPod.

Patients lacking sufficient understanding of the Dutch language and/or participating in concurrent intervention studies were excluded.

### Measurements

During the baseline assessment, sociodemographic, physical, psychological, and behavioral characteristics (eg, age, sex, BMI, smoking status, marital status, working status, level of education [The Netherlands; low: primary school only and lower vocational education, medium: secondary vocational education, and high: higher vocational education and academic education], Charlson Comorbidity Index [CCI], forced expiratory volume in 1 second [FEV_1_] as percentage from the predicted volume, the forced expiratory volume in 1 second to forced vital capacity ratio, GOLD stage based on forced expiratory volume in 1 second % predicted [score of 1-4], occurrence of an exacerbation and exacerbation-related hospitalization in the previous 12 months [33], and the Modified Medical Research Council [mMRC dyspnea scale]) were assessed as described elsewhere [34]. The mMRC dyspnea scale assesses the current severity of dyspnea on a scale of 5 grades (0-4), each describing a level of activity that induces dyspnea, ranging from 0 (“only gets breathless with strenuous exercise”) to 4 (“too breathless to leave the house or breathless when dressing”) [34]. An mMRC score of ≥2 indicates severe dyspnea [1]. The Checklist Individual Strength—subscale subjective fatigue (CIS-Fatigue) was used in which a score of ≥36 points denoted severe fatigue [35]. Anxiety and depression symptoms were assessed using the Hospital Anxiety and Depression Scale (HADS), where a score of >11 points indicates probable symptoms of anxiety/depression [36].

### EMA Symptom Assessment

Electronic EMA questionnaires were completed for 5 consecutive days through an iPod Touch (Apple) with a custom-installed app called PsyMate (version 1.1b). During the baseline assessment, patients received oral instructions on how to use the iPod Touch and were given a printed instruction to take home. Patients were told to carry the iPod Touch with them at all times and keep to their usual day/night routine. Patients had to answer the questions immediately after the beep but no longer than 15 minutes after the beep; otherwise, the questionnaire would be skipped. PsyMate was programmed to beep at 8 random time points between 7:30 AM and 10:30 PM. When receiving the beep, patients had to rate 10 symptoms with a 7-point Likert scale, ranging from 1 (“barely”) to 7 (“very”). A score of 1‐ to 2 is classified as ‘not’, 3‐ to 5 is classified as ‘somewhat’, and 6‐7 is classified as ‘very’. These symptoms included ‘I feel: relaxed; short of breath; energetic; cheerful; insecure; irritated; satisfied; anxious; tired; and mentally fit.’ On the second day, patients were called to ensure everything was going well and to discuss if any problems occurred.

### Physical Activity

During the EMA home monitoring period, patients were asked to wear the ActiGraph GT9X Link 3-axis activity monitor with a sample frequency of 30 Hz for 7 consecutive days. Patients were instructed to wear the ActiGraph on the right hip from the moment they woke up until the moment they went to sleep, keep a normal day/night routine, and remove the ActiGraph if they had to shower or went swimming. During the baseline assessment, the ActiGraph was demonstrated, and printed instructions were provided for patients to take home. Data were recorded in 10-second epochs, and patients’ steps were used to indicate the amount of PA because this is a simple metric and captures the most relevant and problematic daily activity for the majority of patients with COPD [37].

### Sedentary Time

Freedson Adult (1998) activity counts were used to categorize ST. ST was tracked during waking hours and was defined as a period in minutes where the activity counts fell into the sedentary range (0-99) with no interruption [38]. For valid PA data, the wear time per day needed to be a minimum of 8 hours with a minimum of 4 days. These 4 days needed to include at least one weekend day and 3 weekdays [37,39].

### Data Cleaning

If patients completed less than one-third of the total beeps (ie, 13 of 40 beeps), these patients were excluded using R statistical software [40]. If the time between the first and last answer on the EMA questionnaire was longer than 10 minutes, this was considered invalid, and the EMA datapoints were excluded. ActiLife6 software was used to check whether PA and ST data of the same 5 EMA home monitoring days were valid, and this was manually aligned [41]. For all the patients from Nijmegen, the ActiGraph started measuring at 9 AM on the first day because of incorrect initialization, but these patients still have the minimum of 8 hours of wear time on this day. To ensure data validity, ActiGraph wear time was verified 15 and 30 minutes before and after each EMA prompt. The EMA datapoint was excluded using MATLAB if the ActiGraph was not worn during the relevant time windows.

### Data Analysis

**Figure 1.**
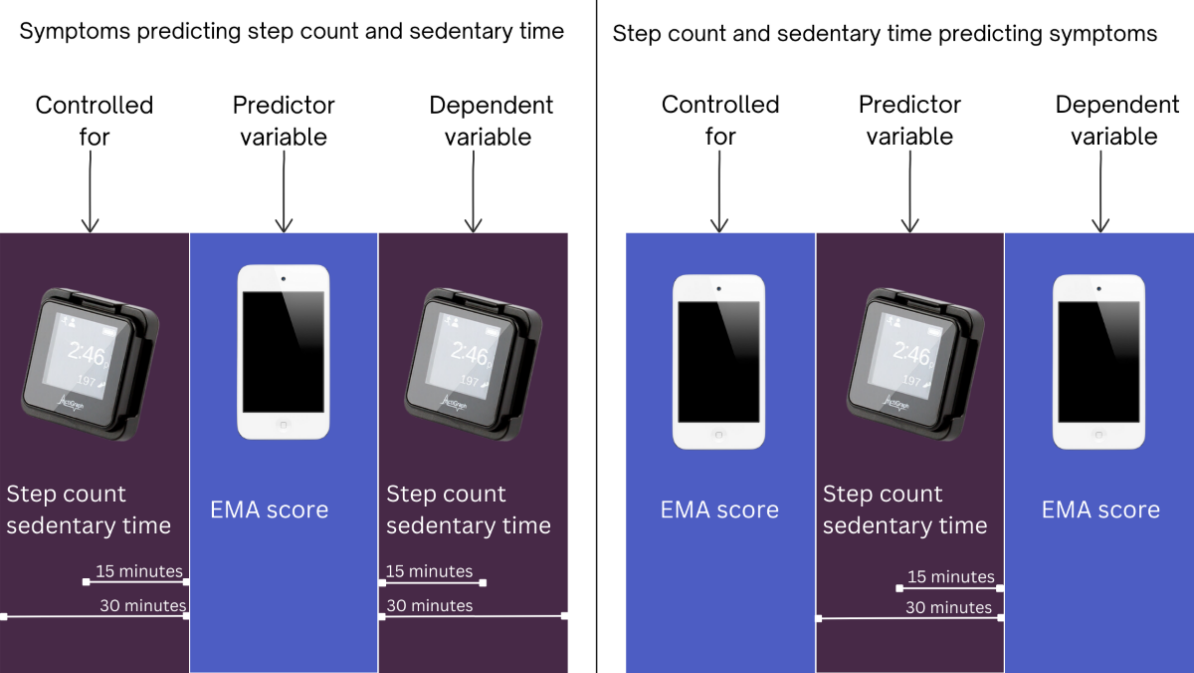
Illustration of the multilevel model methodology. EMA: ecological momentary assessment.

All analyses were performed using SPSS (IBM SPSS Statistics for Windows, version 28.0.1.1; IBM Corp, Armonk, NY, USA). The distribution of data was tested using the Shapiro-Wilk test with a 2-tailed significance of <.05. Descriptive statistics were reported as means and SDs, median and quartiles 1 and 3 (Q1-Q3), percentages, and numbers as appropriate. To examine the direction of the association between steps, ST, and symptoms assessed with EMA, 15- and 30-minute time windows were created before giving the first answer on the EMA questionnaire (pre-EMA) and after giving the last answer (post-EMA), which is in line with previous research [26-28]. The total number of steps and ST within each time window was captured. Data from 11 patients were collected during the COVID-19 pandemic, and patients were measured during all 4 seasons throughout the year, which could potentially influence mood and alter PA and ST patterns. Therefore, based on normality, an independent samples *t* test or Mann-Whitney *U* test was done to compare the average amount of steps and ST during the 5 days between patients measured during COVID-19 and not during COVID-19. Furthermore, as appropriate, a one-way ANOVA or an independent Kruskal-Wallis test was done to compare the average number of steps and ST during the 5 days between the 4 seasons. A priori, a Spearman correlation analysis was run to screen for potential confounders. This was done to determine the relationship between the total number of steps and total ST of the 5 days and between EMA symptom scores and several characteristics, including age, sex, BMI, smoking status, marital status, working status, CCI, GOLD stage, exacerbation history, and exacerbation-related hospitalization in the previous 12 months [8,9,42]. The correlation between time of day with steps, ST, and EMA symptom scores was also tested because previous research showed associations between PA, ST, and time of day [18,43]. Confounders that had a significant correlation (*P*<.05) were used as covariates in the multilevel models. Patient ID was included as a random intercept, specifying that all observations by the same patient would be correlated with each other. Multilevel linear mixed models were performed using the ‘mixed’ command and restricted maximum likelihood estimations with a significance of <.05. While we hypothesized associations specifically for dyspnea and fatigue based on prior literature and clinical relevance, analyses involving the remaining symptoms were exploratory in nature. These additional analyses aimed to identify potential patterns in the data and generate hypotheses for future research. In multilevel model 1, step count 15 and 30 minutes post-EMA was used as the dependent variable, and the EMA score was used as the predictor (Figure 1). This was controlled for the step count 15 and 30 minutes pre-EMA.

In multilevel model 2, the EMA score was used as the dependent variable, and the step count 15 and 30 minutes pre-EMA was used as predictor. This was controlled for the previous EMA score on the same day, so the first EMA score of a day was not corrected for a previous EMA score. In multilevel model 3, ST 15 and 30 minutes post-EMA was used as the dependent variable, and the EMA score was used as the predictor. This was controlled for ST 15 and 30 minutes pre-EMA. In multilevel model 4, the EMA score was used as the dependent variable, and the ST 15 and 30 minutes pre-EMA was used as predictor. This was controlled for the previous EMA score. All 10 symptoms were tested in separate multilevel models.

## Results

### Overview

A total of 60 patients were assessed for eligibility. However, 26 (43%) patients were excluded because of the absence of valid ActiGraph data (n=23) or valid EMA data (n=3). Specifically, a technical error and incorrect initialization of the ActiGraph resulted in 10 (43%) patients lacking any ActiGraph data. In addition, another 10 (43%) patients had ActiGraph data, but they were not valid since seven of these patients had only 1 day of PA measurement, and 3 patients had just 1 day of PA measurement but with less than 4 hours of valid recording. In addition, 3 (13%) patients had a mismatch between the PA and EMA measurement days. Two (67%) patients did not have any EMA data available, and 1 (33%) patient had less than one-third of answered EMA responses. Consequently, 34 patients were included for this analysis.

### Participants

As a lot of patients were excluded, the sociodemographic, physical, psychological, and behavioral characteristics of the included and excluded patients were compared. There were no significant differences in any of these characteristics between the included and excluded patients (Table 1). Of the 34 included patients, 19 were men (56%), 32 (94%) had moderate-to-severe COPD, and almost half had at least one exacerbation in the previous 12 months. Severe dyspnea was reported by 13 of 33 patients (39%), and 15 of 34 patients (44%) experienced severe fatigue. Among the patients, 3 of 33 (9%) and 5 of 33 (15%) had probable symptoms of anxiety and depression, respectively. No statistically significant impact of the COVID-19 pandemic or seasonality on both steps and ST was found (all *P>*.05).

**Table 1.** Baseline characteristics of the included and excluded patients.

Variable^a^	Included (n=34)	N	Excluded (n=26)	N	*P* value
Age (years), mean (SD)	66 (7)	34	69 (6)	26	.19
Male, n (%)	19 (56)	34	19 (73)	26	.17
BMI^b^ (kg/m^2^), median (interquartile range)	24.8 (22.3‐31.5)	34	25.7 (23.7‐28.2)	26	.71
Current smoker, n (%)	4 (12)	34	6 (23)	26	.18
Married/living together, n (%)	21 (62)	34	18 (69)	26	.89
Currently employed, n (%)	6 (18)	33	1 (4)	24	.11
Level of education, n (%)		34		24	.43
Low	6 (18)		5 (21)		
Medium	15 (44)		9 (38)		
High	13 (38)		10 (42)		
CCI^c^ (points), median (interquartile range)	1 (1-2)	34	2 (1-3)	26	.17
FEV_1_^d^ (% predicted), mean (SD)	52 (20)	34	51 (19)	26	.83
FEV_1_/FVC^e^ (%), median (interquartile range)	48 (34‐59)	34	46 (37‐54)	26	.72
GOLD^f^ I/II/III/IV (%)	6/53/29/12	34	8/31/46/15	26	.39
Exacerbation in previous 12 months, n (%)	16 (47)	34	11 (42)	26	.71
Exacerbation-related hospitalization in previous 12 months, n (%)	4 (12)	34	6 (23)	26	.24
mMRC^g^ dyspnea (grade), median (interquartile range)	1 (0‐2)	33	1 (0‐2)	24	.73
Severe dyspnea, n (%)	13 (39)		11 (46)		
CIS-Fatigue^h^ (points), mean (SD)	34 (15)	34	35 (11)	24	.77
Severe fatigue, n (%)	15 (44)		12 (50)		
HADS-anxiety^i^ (points), median (interquartile range)	4 (1-7)	33	7 (3-9)	24	.06
Possible symptoms of anxiety (8-10 points), n (%)	4 (12)		6 (25)		
Probable symptoms of anxiety (≥11 points), n (%)	3 (9)		3 (13)		
HADS-depression**^i^** (points), median (interquartile range)	4 (1-9)	33	4 (2-7)	24	.99
Possible symptoms of depression (8-10 points), n (%)	4 (12)		4 (17)		
Probable symptoms of depression (≥11 points), n (%)	5 (15)		1 (4)		

a Quantitative variables are presented as mean (standard deviation) or median (quartile 1 - quartile 3) for skewed variables.

b BMI: Body Mass Index.

c CCI: Charlson Comorbidity Index.

d FEV1 % predicted: forced expiratory volume in 1 second as percentage from the predicted volume.

e FEV1/FVC %: the forced expiratory volume in 1 second to forced vital capacity ratio.

f GOLD: Global Initiative for Chronic Obstructive Lung Disease.

g mMRC: modified Medical Research Council dyspnea Scale.

h CIS: Checklist Individual Strength.

i HADS: Hospital Anxiety and Depression Scale.

### EMA Symptom Assessment

Of the 1054 EMA datapoints, 5 were excluded because the time between the first and last answer of the EMA questionnaire was longer than 10 minutes, and 1 was excluded because it was sent after 10:30 PM. In addition, 13 EMA datapoints were excluded because patients received the first EMA prompt before 9 AM, whereas the ActiGraph started measuring at 9 AM on the first day, so no ActiGraph data were available. Consequently, 1035 EMA responses were analyzed, resulting in a response rate of 76.1% (1035 completed EMA responses of the 1360 possible EMA responses). The mean response rates of the 34 patients per day were 196 of 272 (72.1%), 221 of 272 (81.3%), 213 of 272 (78.3%), 198 of 272 (72.8%), and 207 of 272 (76.1%). The lowest number of total responses during the 5 days was 18 of 40 (n=1), and the highest number of total responses was 38 of 40 (n=3; Table 2; ).

**Figure 2.**
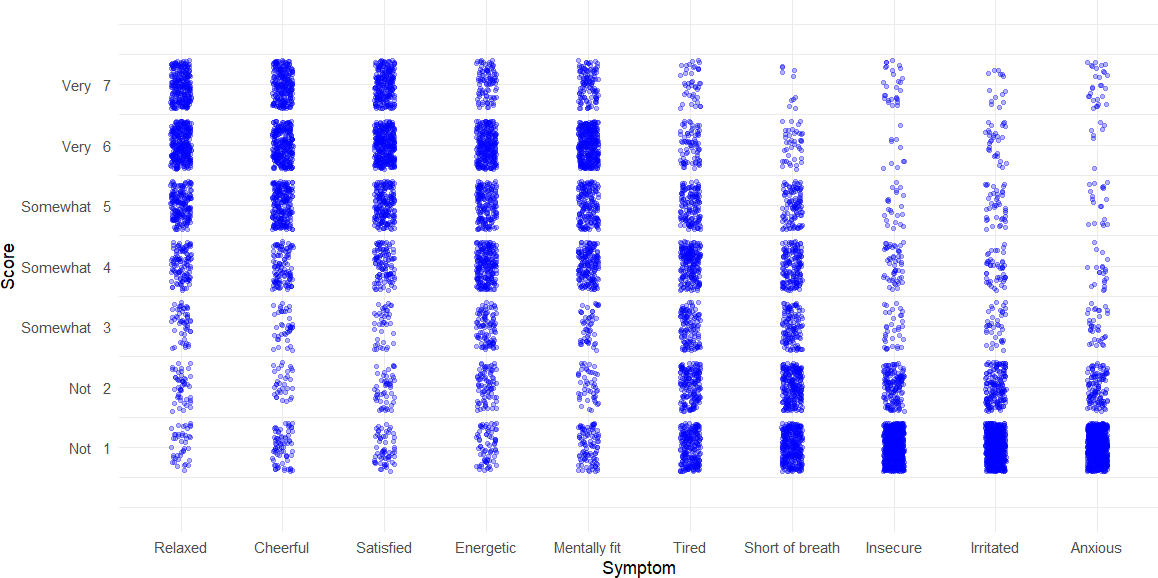
Jittered scatterplot of the EMA-based symptoms scored on a 7-point Likert scale for 5 consecutive days.

The median (Q1-Q3) time of completing the EMA was 1 minute and 1 second (00:48-01:22). During the 5 days, patients generally rated feeling very relaxed, cheerful, and satisfied, while not feeling insecure, irritated, and anxious (Figure 2).

**Table 2.** Total EMA^a^ responses of the 5 days.

Total EMA responses	Patients, n (%)
18	1 (3)
20	1 (3)
22	1 (3)
23	2 (6)
24	2 (6)
27	2 (6)
28	2 (6)
29	1 (3)
30	3 (9)
31	3 (9)
32	3 (9)
33	1 (3)
34	3 (9)
35	3 (9)
36	2 (6)
37	1 (3)
38	3 (9)

a EMA: Ecological Momentary Assessment.

### Physical Activity

During the 5 days, patients had a median (Q1-Q3) of 19 (3-74) steps pre-EMA and 20 (3-67) steps post-EMA in the 15-minute time windows. During the 30-minute time windows, patients had a median (Q1-Q3) of 55 (10-148) steps pre-EMA and 52 (11-139) steps post-EMA.

In the unadjusted models 1, it was found that when patients reported feeling more energetic and more mentally fit, step count was statistically significantly higher 15 minutes post-EMA, and when patients reported feeling more tired, step count was lower 15 and 30 minutes post-EMA (Table 3).

**Table 3.** Associations between symptoms and step count 15 and 30 minutes post–ecological momentary assessment.

Symptom	15-minute coefficient estimate (95% CI)				30-minute coefficient estimate (95% CI)			
	Unadjusted	*P* value	Adjusted^a^	*P* value	Unadjusted	*P* value	Adjusted^a^	*P* value
Relaxed	4.9 (−0.2 to 9.2)	.06	5.1 (0.9 to 10.1)	.046	4.9 (−3.8 to 13.5)	.27	5.8 (−3.6 to 15.2)	.23
Short of breath	−2.6 (−7.6 to 2.5)	.32	0.1 (−5.4 to 5.6)	.97	−5.9 (−14.9 to 3.2)	.20	−1.8 (−12.0 to 8.4)	.72
Energetic	5.9 (1.1 to 10.6)	.02	4.8 (−0.3 to 10.0)	.06	6.6 (−1.9 to 15.1)	.13	4.3 (−5.2 to 13.9)	.37
Cheerful	4.1 (−0.7 to 8.9)	.09	4.0 (−1.1 to 9.1)	.12	6.6 (−1.9 to 15.2)	.13	6.4 (−2.9 to 15.8)	.18
Insecure	2.5 (−3.4 to 8.4)	.40	1.7 (−5.3 to 8.7)	.63	6.4 (−4.2 to 17.0)	.23	6.0 (−7.2 to 19.2)	.37
Irritated	3.6 (−1.8 to 9.1)	.19	2.2 (−3.9 to 8.3)	.49	9.1 (−1.0 to 19.1)	.08	7.4 (−4.1 to 18.9)	.21
Satisfied	2.7 (−2.1 to 7.4)	.27	2.8 (−2.2 to 7.8)	.27	−0.3 (−8.9 to 8.3)	.95	−0.9 (−10.2 to 8.4)	.85
Anxious	2.2 (−4.4 to 8.8)	.52	1.4 (−7.0 to 9.7)	.75	4.1 (−7.5 to 15.8)	.48	3.4 (−12.2 to 19.0)	.67
Tired	−5.4 (−9.9 to −0.8)	.02	−3.9 (−8.4 to 0.7)	.10	−9.8 (−18.0 to −1.5)	.02	−7.4 (−15.9 to 1.1)	.09
Mentally fit	5.0 (0.3 to 9.7)	.04	4.7 (−0.3 to 9.7)	.07	7.6 (−0.7 to 15.9)	.07	7.0 (−2.0 to 16.1)	.13

a Adjusted for time of day, working status, GOLD stage, exacerbation history, and exacerbation-related hospitalization in the previous 12 months.

The total number of steps taken during the 5 days was positively correlated with being currently employed (*r*=0.38; *P*=.03) and negatively correlated with GOLD stage (*r*=−0.53; *P*<.001), exacerbation history (*r*=−0.41; *P*=.02), and exacerbation-related hospitalization in the previous 12 months (*r*=−0.36; *P*=.04). Furthermore, steps were negatively correlated with time of day (*r*=−0.12; *P*<.001). When adjusting for these significant confounders, no significant associations were found in models 1 between symptoms and step count 30 minutes post-EMA. Only feeling relaxed had a positive relationship with step count 15 minutes post-EMA. That is, for a 1-unit increase in feeling relaxed, the expected value of step count 15 minutes post-EMA increases by 5.1 steps.

Feeling short of breath was positively correlated with GOLD stage (*r*=0.44; *P*=.009), whereas feeling energetic was negatively correlated with GOLD stage (*r*=−0.35; *P*=.046). Feeling cheerful and satisfied was both negatively correlated with being a current smoker (*r*=−0.38*; P*=.03; and *r*=−0.41; *P*=.02), and feeling irritated was negatively correlated with CCI points (*r*=−0.34; *P*=.049). Furthermore, feeling relaxed was positively correlated with time of day (*r*=0.12; *P*<.001), whereas feeling irritated was negatively correlated with time of day (*r*=−0.07; *P*=.04).

In both the unadjusted and adjusted models 2, it was found that a higher step count during a 15- and 30-minute time window pre-EMA was associated with feeling less relaxed and feeling more short of breath and tired (Table 4).

**Table 4.** Associations between step count 15 and 30 minutes pre–ecological momentary assessment and symptoms.

Symptom	15-minute coefficient estimate (95% CI)				30-minute coefficient estimate (95% CI)			
	Unadjusted	*P* value	Adjusted	*P* value	Unadjusted	*P* value	Adjusted	*P* value
Relaxed	−6.4×10^−4^ (−1.1×10^4^ to −2.0×10^−4^)	.005	−5.2×10^−4^ (−9.7×10^−4^ to −7.0×10^−5^)^a^	*.*02	−3.9×10^−4^ (−6.2×10^−4^ to −1.5×10^−4^)	.001	−3.2×10^−4^ (−5.6×10^−4^ to −7.9×10^−5^)^a^	.009
Short of breath	8.4×10^−4^ (4.6×10^−4^ to 1.2×10^−3^)	<.001	8.5×10^−4^ (4.7×10^−4^ to 1.2×10^−3^)^b^	<.001	4.6×10^−4^ (2.6×10^−4^ to 6.6×10^−4^)	<.001	4.6×10^−4^ (2.6×10^−4^ to 6.6×10^−4^)^b^	*<*.001
Energetic	2.3×10^−4^ (−1.8×10^−4^ to 6.3×10^−4^)	.27	2.2×10^−4^ (−1.8×10^−4^ to 6.2×10^−4^)^b^	.29	1.6×10^−4^ (−5.0×10^−5^ to 3.7×10^−4^)	.13	1.6×10^−4^ (−5.4×10^−5^ to 3.7×10^−4^)^b^	.14
Cheerful	−5.1×10^−5^ (−4.3×10^−4^ to 3.3×10^−4^)	.79	−4.0×10^−5^ (−4.2×10^−4^ to 3.4×10^-4^)^c^	.84	−4.5×10^−5^ (−2.5×10^−4^ to 1.5×10^−4^)	.66	-4.0×10^-5^ (−2.4×10^−4^ to 1.6×10^−4^)^c^	.69
Insecure	2.3×10^−4^ (−7.2×10^−5^ to 5.3×10^−4^)	.14	N/A^e^		8.8×10^−5^ (−7.2×10^−5^ to 2.5×10^−4^)	.28	N/A	
Irritated	3.0×10^−4^ (−7.5×10^−5^ to 6.8×10^−4^)	.12	2.6×10^−4^ (1.2×10^−4^ to 6.4×10^−4^)^a^^,d^	.18	1.0×10^−4^ (−9.3×10^−5^ to 3.0×10^−4^)	.30	8.0×10^−5^ (−1.2×10^−4^ to 2.8×10^−4^)^a^^,d^	.44
Satisfied	−3.6×10^−4^ (−7.7×10^−4^ to 5.5×10^−5^)	.09	−3.4×10^−4^ (−7.5×10^−4^ to 6.8×10^−5^)^c^	.10	−1.5×10^−4^ (−3.7×10^−4^ to 6.7×10^−5^)	.17	−1.5×10^−4^ (−3.6× 10^−4^ to 7.3×10^−5^)^c^	.19
Anxious	1.3×10^−4^ (−1.3×10^−4^ to 3.9×10^−4^)	.33	N/A		5.4×10^−5^ (−8.5×10^−5^ to 1.9×10^−4^)	.45	N/A	
Tired	5.1×10^−4^ (7.2×10^5^ to 9.4×10^-4^)	.02	N/A		2.9×10^−4^ (5.3×10^−5^ to 5.2×10^−4^)	.02	N/A	
Mentally fit	−1.2×10^−4^ (−4.9×10^−4^ to 2.4×10^−4^)	.51	N/A		−6.0×10^-5^ (−2.5×10^−4^ to 1.3×10^−4^)	.54	N/A	

a Adjusted for time of day.

b Adjusted for Global Initiative for Chronic Obstructive Lung Disease stage.

c Adjusted for smoking status.

d Adjusted for Charlson Comorbidity Index.

e Not applicable.

### Sedentary Time

Patients, on average (SD), spent 314 (116) minutes sedentary per patient per day during waking hours. During the 5 days, patients had a median (Q1-Q3) of 9 (3-13) minutes ST pre-EMA and 9 (4-13) minutes post-EMA in the 15-minute time windows. During the 30-minute time windows, patients had a median (Q1-Q3) of 18 (7-24) minutes ST pre-EMA and 18 (9-24) minutes post-EMA.

The total ST during the 5 days was positively correlated with BMI (*r*=0.36; *P*=.04) and negatively correlated with being currently employed (*r*=−0.39; *P*=.03). In addition, ST was positively correlated with time of day (*r*=0.24; *P*<.001). In both the unadjusted and adjusted models 3, no significant associations were found between symptoms and 15 or 30 minutes post-EMA ST (Table 5).

In the unadjusted models 4, it was found that a higher ST during a 15- and 30-minute time window pre-EMA was associated with feeling more anxious (Table 6).

**Table 5.** Associations between symptoms and sedentary time 15 and 30 minutes post–ecological momentary assessment.

Symptom	15-minute coefficient estimate (95% CI)				30-minute coefficient estimate (95% CI)			
	Unadjusted	*P* value	Adjusted^a^	*P* value	Unadjusted	*P* value	Adjusted^a^	*P* value
Relaxed	−6.0 (−16.9 to 4.9)	.28	−8.8 (−20.4 to 2.8)	.14	−2.6 (−23.4 to 18.1)	.80	−6.4 (−28.5 to 15.8)	.57
Short of breath	−0.9 (−13.0 to 11.1)	.88	−1.3 (−14.3 to 11.6)	.84	−7.1 (−30.1 to 16.0)	.55	−5.9 (−30.8 to 19.0)	.64
Energetic	4.0 (−7.3 to 15.3)	.49	5.0 (−7.0 to 16.9)	.42	5.6 (−16.1 to 27.3)	.61	5.1 (−17.9 to 28.0)	.67
Cheerful	−3.5 (−14.8 to 7.9)	.55	−3.1 (−15.3 to 9.2)	.62	−5.0 (−26.9 to 16.9)	.65	−6.3 (−29.9 to 17.2)	.60
Insecure	−4.9 (−18.9 to 9.1)	.49	−5.5 (−22.0 to 11.0)	.52	−2.8 (−30.5 to 24.9)	.84	0.8 (−32.1 to 33.6)	.96
Irritated	6.3 (−6.4 to 18.9)	.33	8.9 (−5.1 to 22.9)	.21	8.6 (−15.9 to 33.2)	.49	14.8 (−12.5 to 42.0)	.29
Satisfied	−5.9 (−17.0 to 5.2)	.29	−6.9 (−18.7 to 5.0)	.26	−7.4 (−28.7 to 13.9)	.50	−10.0 (−32.8 to 12.7)	.39
Anxious	7.3 (−8.7 to 23.2)	.37	11.6 (−8.6 to 31.8)	.26	13.5 (−17.4 to 44.4)	.39	26.1 (-12.6 to 64.8)	.19
Tired	−3.2 (−13.7 to 7.4)	.55	−4.4 (−15.6 to 6.8)	.44	−6.2 (−26.5 to 14.1)	.55	−5.9 (−27.3 to 15.6)	.59
Mentally fit	−3.1 (−14.6 to 8.4)	.59	−2.6 (−14.9 to 9.6)	.67	−1.1 (−23.2 to 21.0)	.92	−1.7 (−25.2 to 21.9)	.89

a Adjusted for time of day, BMI, and working status.

**Table 6.** Associations between sedentary time 15 and 30 minutes pre–ecological momentary assessment and symptoms.

Symptom	15-minute coefficient estimate (95% CI)				30-minute coefficient estimate (95% CI)			
	Unadjusted	*P* value	Adjusted	*P* value	Unadjusted	*P* value	Adjusted	*P* value
Relaxed	1.9×10^−4^ (−6.6×10^−5^ to 4.4×10^−4^)	.15	1.5×10^−4^ (−1.1×10^−4^ to 4.0× 10^−4^)^a^	.26	9.4×10^−5^ (−4.5× 10^−5^ to 2.3×10^−4^)	.18	6.7×10^−5^ (7.2×10^−5^ to 2.1×10^−4^)^a^	.34
Short of breath	−1.3×10^-4^ (−3.4× 10^−4^ to 9.3×10^−5^)	.26	−1.3×10^−4^ (−3.5× 10^−4^ to 8.8×10^−5^)^b^	.24	−9.2×10^−5^ (−2.1× 10^−4^ to 2.7×10^−5^)	.13	−9.4×10^−5^ (−2.1× 10^−4^ to 2.4×10^−5^)^b^	.12
Energetic	−4.9×10^−5^ (−1.8×10^−4^ to 2.8× 10^−4^)	.67	5.1×10^-5^ (−1.8×10^−4^ to 2.8×10^−4^)^b^	.66	1.5×10^−5^ (−1.4×10^−4^ to 1.1×10^−4^)	.81	−1.3×10^−5^ (−1.4×10^−4^ to 1.1× 10^−4^)^b^	.83
Cheerful	1.2×10^−4^ (−6.9×10^−5^ to 3.4×10^−4^)	.28	1.1×10^−4^ (−1.0×10^−4^ to 3.3×10^−4^)^c^	.30	3.9×10^−5^ (−7.9×10^−5^ to 1.6×10^−4^)	.52	3.5×10^−5^ (−8.2×10^−5^ to 1.5×10^−4^)^c^	.56
Insecure	1.4×10^−4^ (−3.3×10^−5^ to 3.1×10^−4^)	.11	N/A^e^		7.6×10^−5^ (−1.5×10^−5^ to 1.7×10^−4^)	.10	N/A	
Irritated	4.6×10^−5^ (−1.7×10^−4^ to 2.6×10^−4^)	.69	6.0×10^−5^ (−1.5×10^−4^ to 2.7×10^−4^)^a^^,d^	.58	4.8×10^−5^ (−6.7×10^−5^ to 1.6×10^−4^)	.41	6.7×10^−5^ (−5.9× 10^−5^ to 1.7×10^−4^)^a^^,d^	.34
Satisfied	1.2×10^−4^ (−1.2×10^−4^ to 3.5×10^−4^)	.34	1.1×10^−4^ (−1.3×10^−4^ to 3.5×10^−4^)^c^	.36	6.0×10^−5^ (−7.3× 10^−5^ to 1.8×10^−4^)	.39	5.2×10^−5^ (−7.6×10^−5^ to 1.8×10^−4^)^c^	.42
Anxious	1.7×10^−4^ (1.7×10^−5^ to 3.2×10^−4^)	*.*03	N/A		8.5×10^−5^ (2.5×10^-6^ to 1.7×10^−4^)	.04	N/A	
Tired	−1.9×10^−4^ (−4.4× 10^−4^ to 5.6×10^−5^)	.13	N/A		−8.0×10^−5^ (−2.2× 10^−4^ to 5.5×10^−5^)	.25	N/A	
Mentally fit	3.7×10^−5^ (−1.7×10^−4^ to 2.5×10^−4^)	.73	N/A		1.4×10^-6^ (−1.2×10^−4^ to 1.1×10^−4^)	.99	N/A	

a Adjusted for time of day.

b Adjusted for Global Initiative for Chronic Obstructive Lung Disease stage.

c Adjusted for smoking status.

d Adjusted for Charlson Comorbidity Index.

e Not applicable.

## Discussion

### Principal Findings

To the best of our knowledge, this is the first study to assess the direction of the association between PA and ST measured with accelerometry and physical and psychological symptoms measured with EMA in patients with mild-to-very severe COPD. This study showed that feeling relaxed was positively associated with an increased step count shortly after completing the EMA. A higher step count before EMA completion was associated with feeling less relaxed, more short of breath, and tired. These findings suggest a potential bidirectional relationship between PA and psychological well-being. Furthermore, our results indicate that ST preceding EMA completion was associated with increased feelings of anxiety, underscoring the potential adverse effect of prolonged ST on psychological well-being in patients with COPD.

### Steps

In this study, feeling more relaxed was the sole predictor for the number of steps 15 minutes post-EMA, after adjusting for relevant confounders. While this effect size may appear modest, it should be interpreted in the context of the overall low step counts observed in this population. Given the median step counts of 19 to 20 steps per 15-minute window, even small increases may reflect meaningful changes in short-term activity patterns. Even minor increases in PA can have positive effects on symptoms, functional status, and quality of life in patients with COPD [13,14]. In contrast, a previous study in healthy older adults found no significant associations between feeling calm/relaxed and subsequent PA, nor between positive (happy and cheerful) and negative (anxious, stress, sad, and angry) affect and subsequent PA 15 and 30 minutes post-EMA [44]. Feeling more energetic was associated with increased PA for both 15- and 30-minute time windows in low-active adults [27]. In this study, feeling more energetic was associated with a higher step count 15 minutes post-EMA only in the unadjusted model. Contradicting results are found regarding the relationship between fatigue and PA. One study found no relationship between fatigue and PA in the 15 and 30 minutes post-EMA in low-active adults [27]. Conversely, other studies involving healthy older adults and patients receiving peritoneal dialysis demonstrated that higher fatigue severity and diminished energy were associated with reduced subsequent PA in the 15, 30, 60, and 120 minutes post-EMA [28,45]. These results suggest that higher fatigue results in lower probability of being physically active and in fewer minutes of subsequent PA [28,45]. In this study, feeling more tired was associated with a lower step count 15 and 30 minutes post-EMA only in the unadjusted models. Confounders such as employment status, GOLD stage, exacerbation history, and exacerbation-related hospitalizations might exert a detrimental influence on patients’ energy and tiredness, potentially disrupting the relationship with engaging in PA [46,47]. Prior studies showed a positive relationship between improved mental state and engaging in PA in patients with COPD [48]. In this study, feeling more mentally fit was associated with a higher step count 15 minutes post-EMA only in the unadjusted model.

In this study, a higher step count 15 and 30 minutes pre-EMA was associated with feeling less relaxed, more short of breath, and tired. Previously, Hevel et al found no significant associations between step count and subsequent positive (happy and cheerful) and negative (anxious, stressed, sad, and angry) affect in healthy older adults [44]. In contrast, previous studies in nonactive adults and patients receiving peritoneal dialysis found that more PA minutes 15 and 30 minutes pre-EMA were associated with feeling more energetic, positive mood, and lower fatigue severity [27,28,44]. These studies generally demonstrate positive psychological effects after PA. Our findings deviate from these results, potentially because patients with COPD have different experiences with performing PA [37,49]. Physical barriers limit the ability of patients with COPD to engage in PA [8]. Patients frequently report that dyspnea, fatigue, and PA-induced coughing are distressing and discourage being physically active, potentially creating a vicious circle where symptoms discourage activity and inactivity exacerbates symptoms [7,8,14,49]. Consequently, increasing PA may exacerbate symptoms, leading to reduced feelings of relaxation and feeling more short of breath and tired [37].

### Sedentary Time

In this study, no significant associations were found between symptoms and subsequent ST. The relationship between symptoms and ST using EMA has been understudied. Prior studies suggest that psychological symptoms such as anxiety, depressed mood, and dyspnea may generally contribute to increased ST in patients with COPD [9,50]. However, these effects may not be directly observed or exist in short-term analysis.

In this study, higher ST was linked to increased feelings of anxiety for both 15- and 30-minute time windows. Only a very small proportion of the study population had probable symptoms of anxiety according to the HADS. This underscores the significant impact of ST on anxiety, aligning with previous research [7].

### Methodological Considerations

The current findings need to be interpreted in the light of the number of comparisons that were made in this study. Nonetheless, multiple findings in the same direction, rather than a single statistically significant result, suggest that these are not due to chance alone. Moreover, ‘Bonferroni adjustments are at best unnecessary and, at worst, deleterious to sound statistical inference’ [51]. This study revealed that EMA through a mobile app was feasible for symptom assessment in technically proficient patients with COPD. Only 3 of 34 patients had insufficient EMA data, and the average response rate (76.1%) is consistent with other feasibility studies exploring the use of EMA in patients with COPD and other clinical populations [26,28,52]. Random time intervals for the EMA assessment prevented patients from anticipating and adjusting their behavior. However, by using time windows surrounding the randomized time intervals of the EMA prompts, instead of examining naturally occurring beginning and ends of taking steps and ST, only partial PA sessions may have been captured. Although data showed a clear variability in step count day to day, we do not expect this to impact the findings as we examined associations between changes in PA, ST, and changes in EMA. The ActiGraph, generally valid and responsive for PA measurements in patients with COPD, had a technical error that invalidated data from 23 patients, reducing the study sample size [37]. The relatively small study sample size and the patients being technically proficient limit the generalizability of our findings. Furthermore, the analyses for symptoms beyond dyspnea and fatigue were exploratory in nature. As these associations were not hypothesized a priori, findings for symptoms such as mental fitness or cheerfulness should be interpreted with caution. Although 1035 EMA scores were related to PA and ST measurements, a larger sample of patients with COPD should be included in future research to test the generalizability and replicability of our findings, including longitudinal follow-up.

### Clinical Implications and Future Directions

Future research is necessary to examine the complex associations between PA, ST, and symptoms in patients with COPD, as many external factors could have exerted an influence on patients’ ability to perform PA, such as weather and social context [9,37,49,53]. This study presents findings that contradict previous research, particularly regarding the effects of PA on mood and energy. While it is plausible that patients with COPD experience PA differently due to physical limitations, the study does not provide definitive answers about the underlying reasons for these discrepancies. The absence of a deeper exploration into these mechanisms highlights the need for further research. Future studies should aim to investigate potential explanations, including physiological, psychological, and contextual factors, to better understand how patients with COPD respond to PA and what drives these differences [27].

### Conclusions

To conclude, a bidirectional association of feeling relaxed with engaging in PA was observed. More PA pre-EMA seemed to result in adverse physical states, including feeling more short of breath and tired. Furthermore, a longer ST pre-EMA seemed to result in an increased feeling of anxiety. Overall, these findings emphasize the complex relationships between PA, ST, and symptoms in the daily lives of patients with COPD.

## Abbreviations

COPD: chronic obstructive pulmonary disease
EMA: ecological momentary assessment
FEV_1_: forced expiratory volume in 1 second
mMRC: Modified Medical Research Council
PA: physical activity
ST: sedentary time
